# Supplementary material for: Spontaneous behavior predicts colony membership, individual identity, and rank in naked mole-rat societies
Source: bioRxiv. 2026 Oct 2:2026.09.27.754730. Preprint. [Version 1] doi: 10.64898/2026.09.27.754730 (PMC13648485; doi:10.64898/2026.09.27.754730)
Supplement: Supplementary file 1 [file NIHPP2026.09.27.754730v1-supplement-4.pdf]

bioRxiv preprint doi: https://doi.org/10.64898/2026.09.27.754730; this version posted October 2, 2026. The copyright holder for this preprint (which was not certified by peer review) is the author/funder, who has granted bioRxiv a license to display the preprint in perpetuity. It is made available under a CC-BY 4.0 International license.

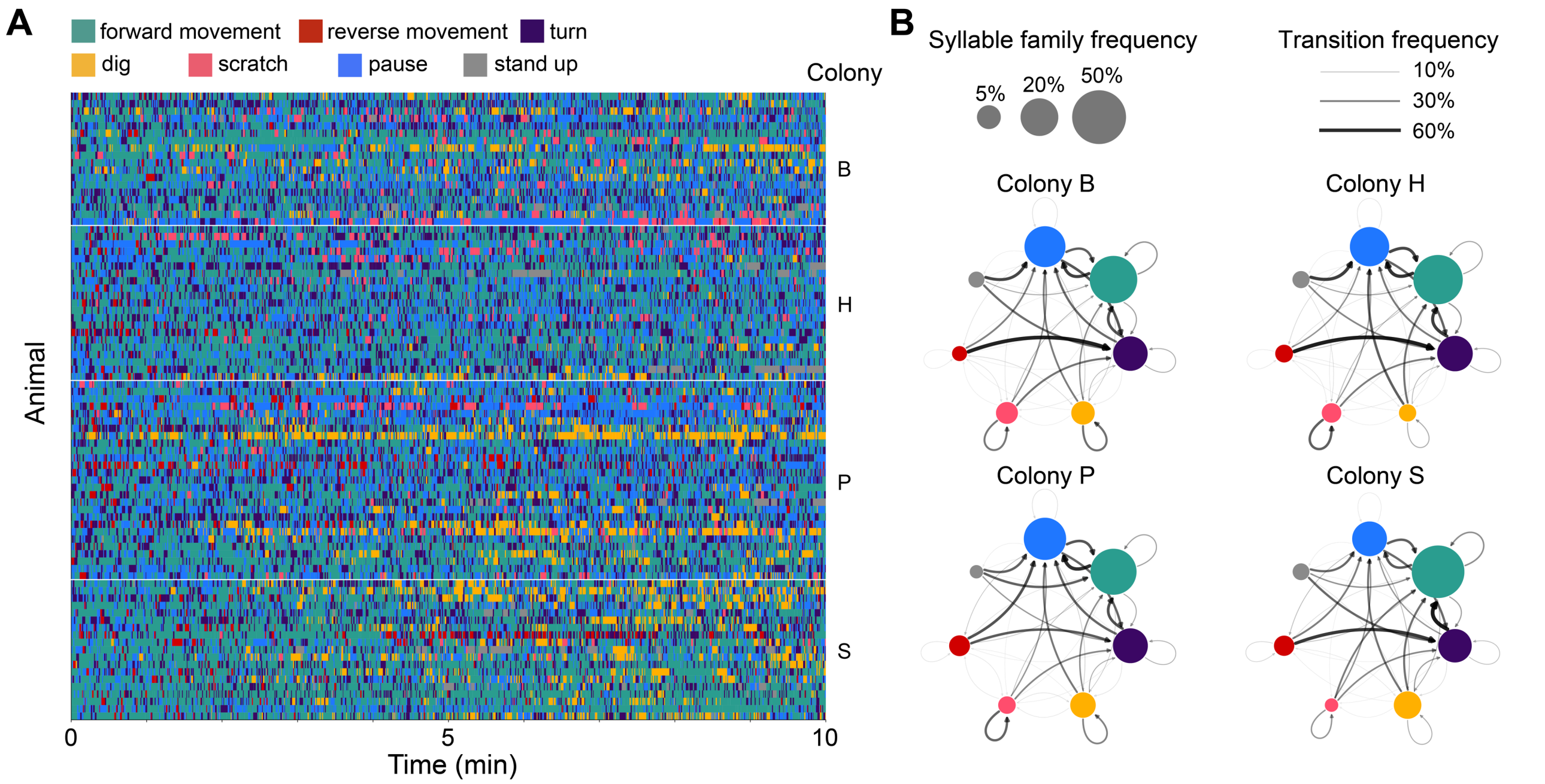

Supplementary Figure S1. Additional details of spontaneous behavioral syllables, related to Figure 1. (**A**) Ethogram of expressed syllables in 10-minute recordings from 85 naked mole-rats in four colonies. Colors indicate syllable families. (**B**) Transition graphs of syllable families across recordings of the four colonies.

bioRxiv preprint doi: https://doi.org/10.64898/2026.09.27.754730; this version posted October 2, 2026. The copyright holder for this preprint (which was not certified by peer review) is the author/funder, who has granted bioRxiv a license to display the preprint in perpetuity. It is made available under a CC-BY 4.0 International license.

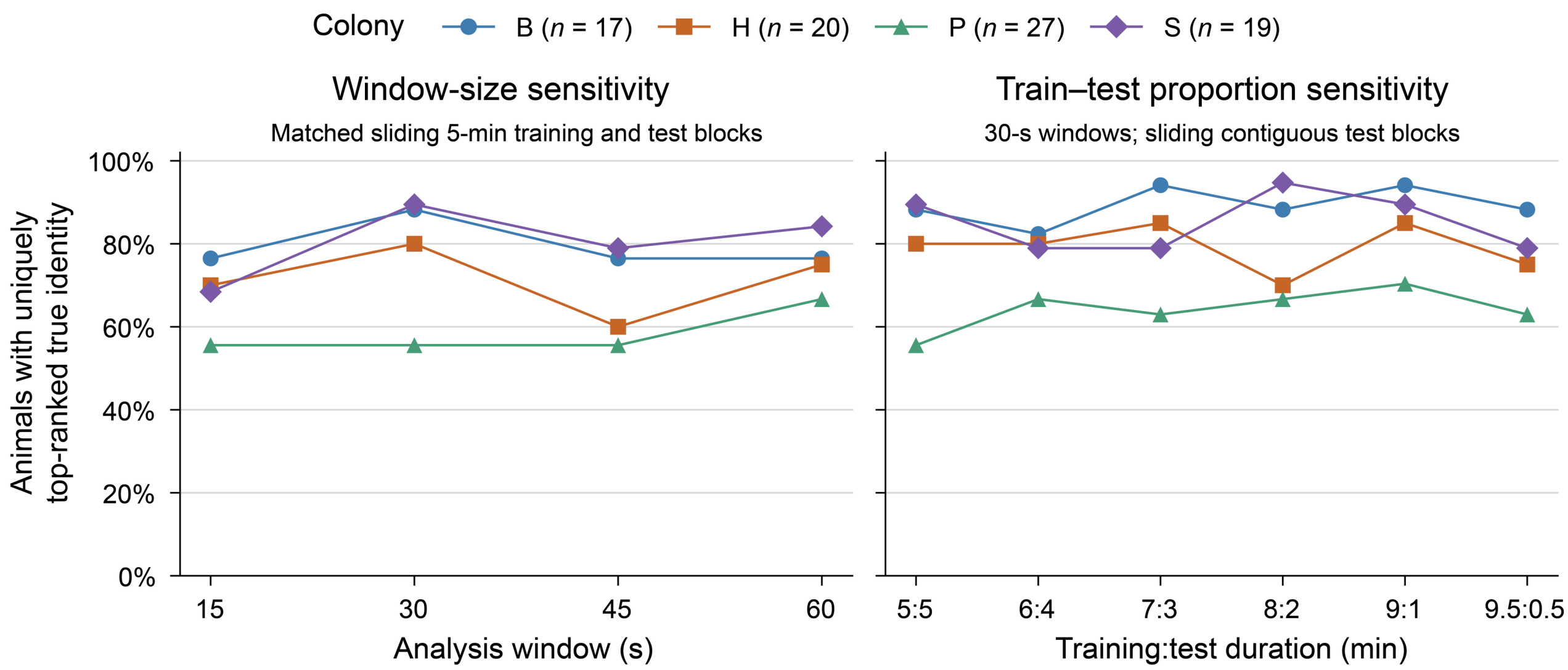

Supplementary Figure S2. Individual identity prediction is robust to analysis window size and training–test ratio. (Left) Percentage of animals for which the true identity received more predictions than any other identity using analysis windows of 15, 30, 45, or 60 seconds. Classifiers were evaluated using matched sliding 5-minute training and test blocks. (Right) Percentage of animals with a uniquely top-ranked true identity across different training durations, using 30-second analysis windows and sliding contiguous test blocks. Colors and symbols indicate colony; sample sizes are shown in the key. Identity prediction remained above colony-specific (1/*N*) chance under every condition (all Bonferroni-adjusted $P < 0.0001$, one-sided exact binomial tests).

bioRxiv preprint doi: https://doi.org/10.64898/2026.09.27.754730; this version posted October 2, 2026. The copyright holder for this preprint (which was not certified by peer review) is the author/funder, who has granted bioRxiv a license to display the preprint in perpetuity. It is made available under a CC-BY 4.0 International license.

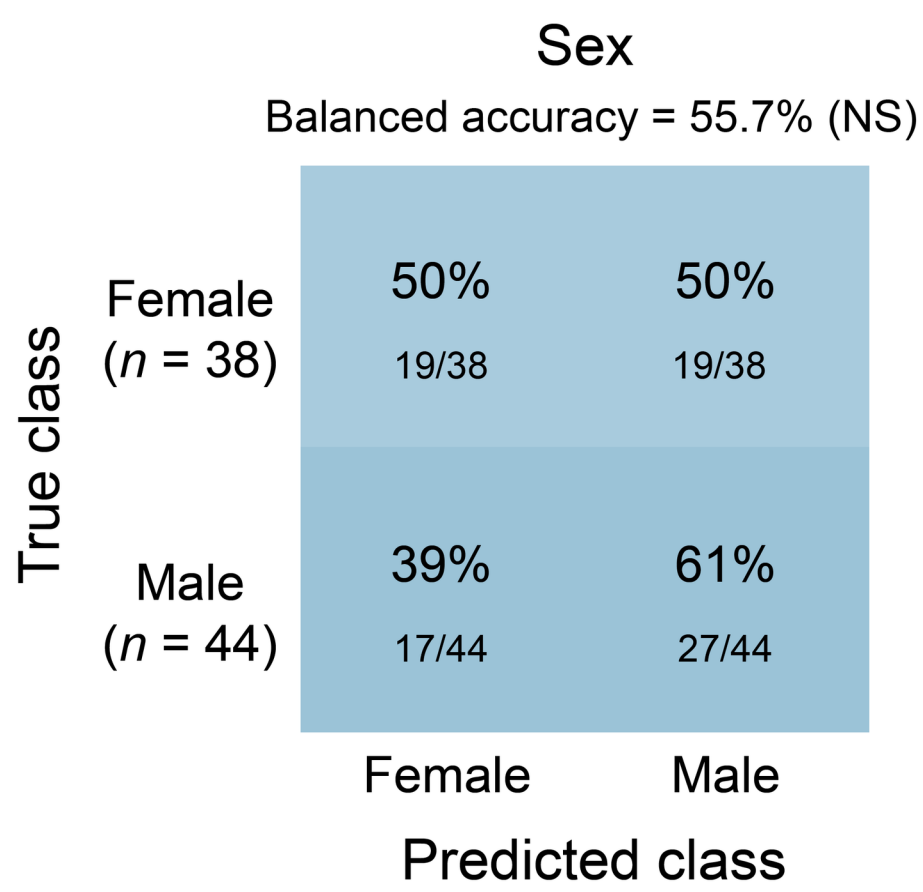

Supplementary Figure S3. Spontaneous behavior did not predict sex. Row-normalized confusion matrix showing leave-one-animal-out sex predictions pooled across colonies. Random forest classifiers using syllable-frequency and behavioral-summary features were trained and evaluated separately within each colony, and the held-out predictions were pooled across colonies for visualization. These sex classifications did not exceed chance in any colony (all Holm-adjusted $P > 0.05$; one-sided within-colony label-permutation tests, 1,000 permutations). Cells show the percentage of animals in each true class assigned to each predicted class; fractions indicate the corresponding number of animals relative to the true-class total. The pooled balanced accuracy was 55.7%; NS, not significant.

bioRxiv preprint doi: https://doi.org/10.64898/2026.09.27.754730; this version posted October 2, 2026. The copyright holder for this preprint (which was not certified by peer review) is the author/funder, who has granted bioRxiv a license to display the preprint in perpetuity. It is made available under a CC-BY 4.0 International license.

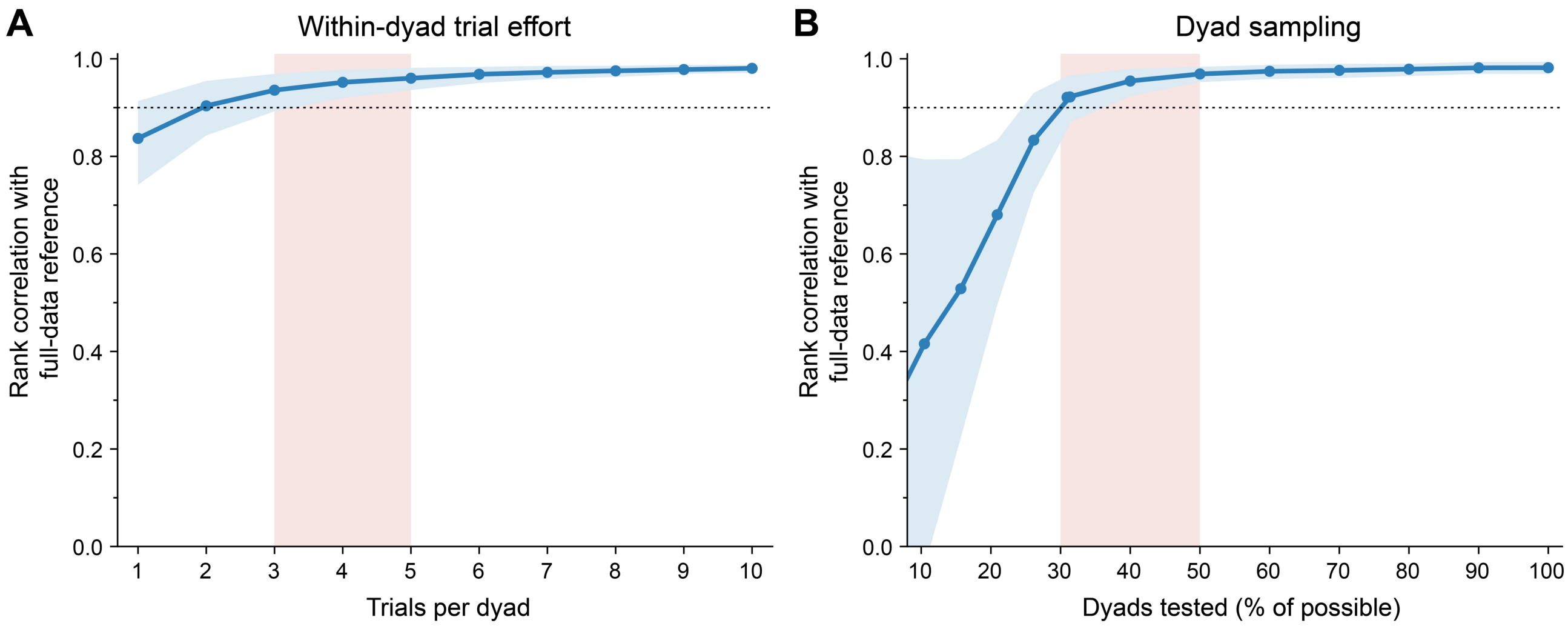

Supplementary Figure S4. Sparse adaptive sampling efficiently recovers colony-wide dominance rank. Exhaustive, repeated dyad testing to rank animals becomes prohibitive as colony size increases. We therefore asked whether colony-wide rank could be recovered without compromising accuracy by testing fewer dyads and fewer trials per dyad. We first established a full-data reference by exhaustively testing colony B, completing 10 trials for all 153 possible dyads among 18 animals. We then retrospectively subsampled this dataset and compared the inferred ranks with the full-data rank order. (**A**) Rank correlation with the full-data reference at 40% dyad coverage when each dyad was tested for a fixed 1–10 trials. These results motivated a stopping rule in which testing ended after a 3–0 outcome but otherwise continued to five trials. (**B**) Rank correlation with the full-data reference as a function of the proportion of possible dyads using this stopping rule. In both panels, blue lines and points indicate means, and blue shading denotes the 10th–90th percentiles across 1,000 simulations per condition. Pale red bands indicate the selected operating ranges of 3–5 trials per dyad and 30%–50% dyad coverage, which we subsequently used to rank colonies H, P, and S. Black dotted lines indicate a rank correlation of 0.9.

bioRxiv preprint doi: https://doi.org/10.64898/2026.09.27.754730; this version posted October 2, 2026. The copyright holder for this preprint (which was not certified by peer review) is the author/funder, who has granted bioRxiv a license to display the preprint in perpetuity. It is made available under a CC-BY 4.0 International license.

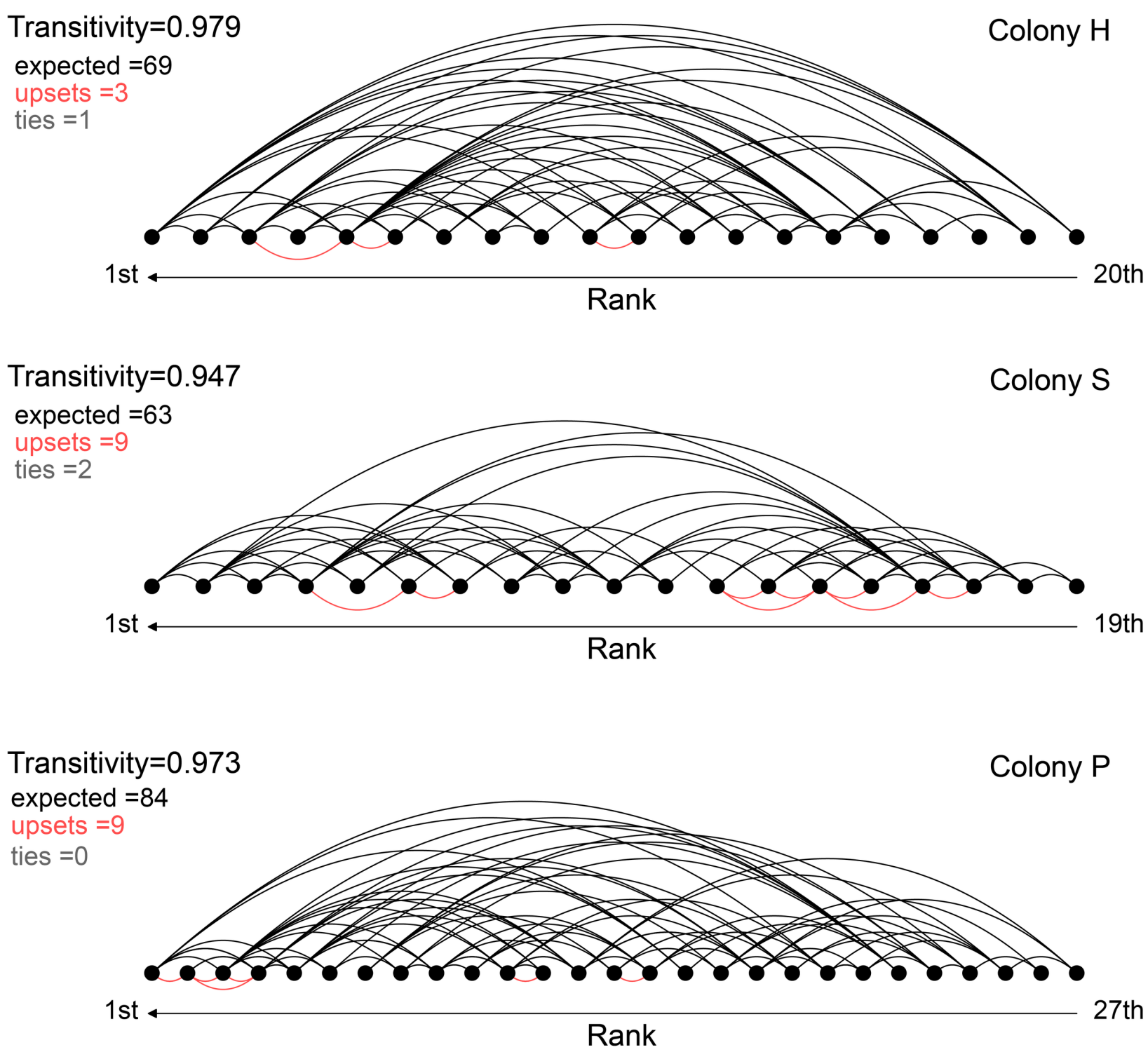

Supplementary Figure S5. Tube-test dominance hierarchies are highly transitive across colonies, related to Figure 3. Rank-ordered dominance networks are shown for colony H (top), colony S (middle), and colony P (bottom). Each node represents an animal, ordered from highest to lowest rank from left to right. Black arcs indicate expected outcomes in which the higher-ranked animal won the dyad, whereas red arcs indicate upsets. Tied dyads are not connected. All three colonies exhibited highly transitive hierarchy structures (P < 0.001 for all colonies by permutation tests; see Methods).

bioRxiv preprint doi: https://doi.org/10.64898/2026.09.27.754730; this version posted October 2, 2026. The copyright holder for this preprint (which was not certified by peer review) is the author/funder, who has granted bioRxiv a license to display the preprint in perpetuity. It is made available under a CC-BY 4.0 International license.

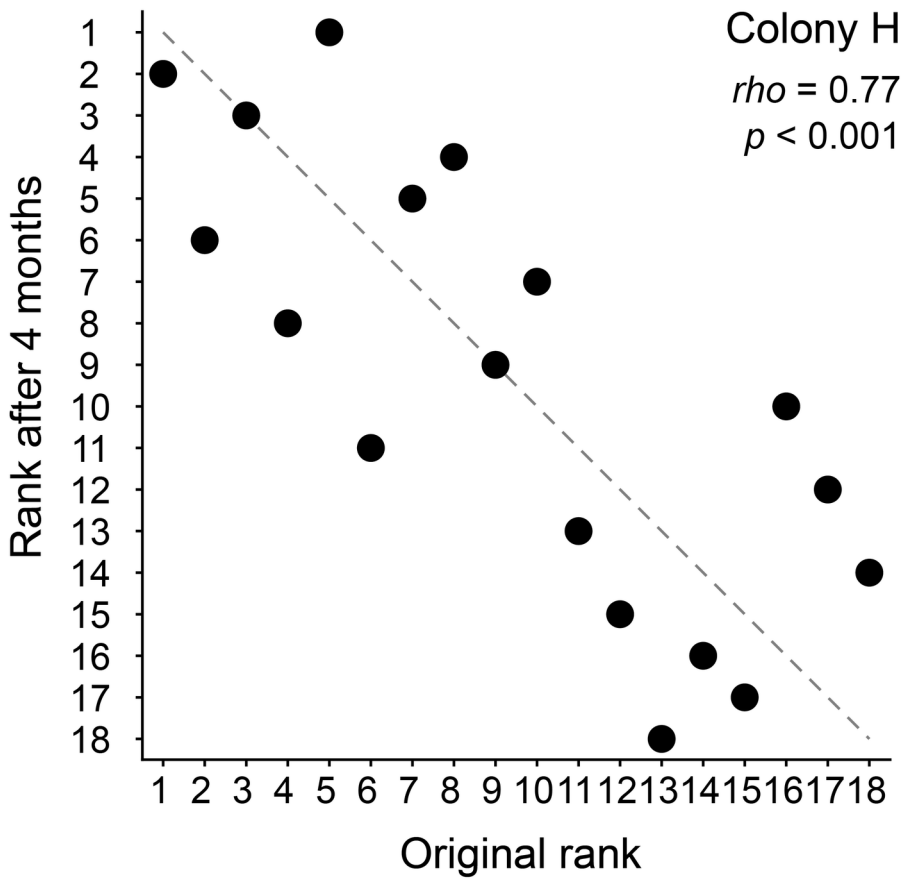

Supplementary Figure S6. Rank remained stable over 4 months in colony H. *rho* and *P*, Spearman's rank correlation test.

bioRxiv preprint doi: https://doi.org/10.64898/2026.09.27.754730; this version posted October 2, 2026. The copyright holder for this preprint (which was not certified by peer review) is the author/funder, who has granted bioRxiv a license to display the preprint in perpetuity. It is made available under a CC-BY 4.0 International license.

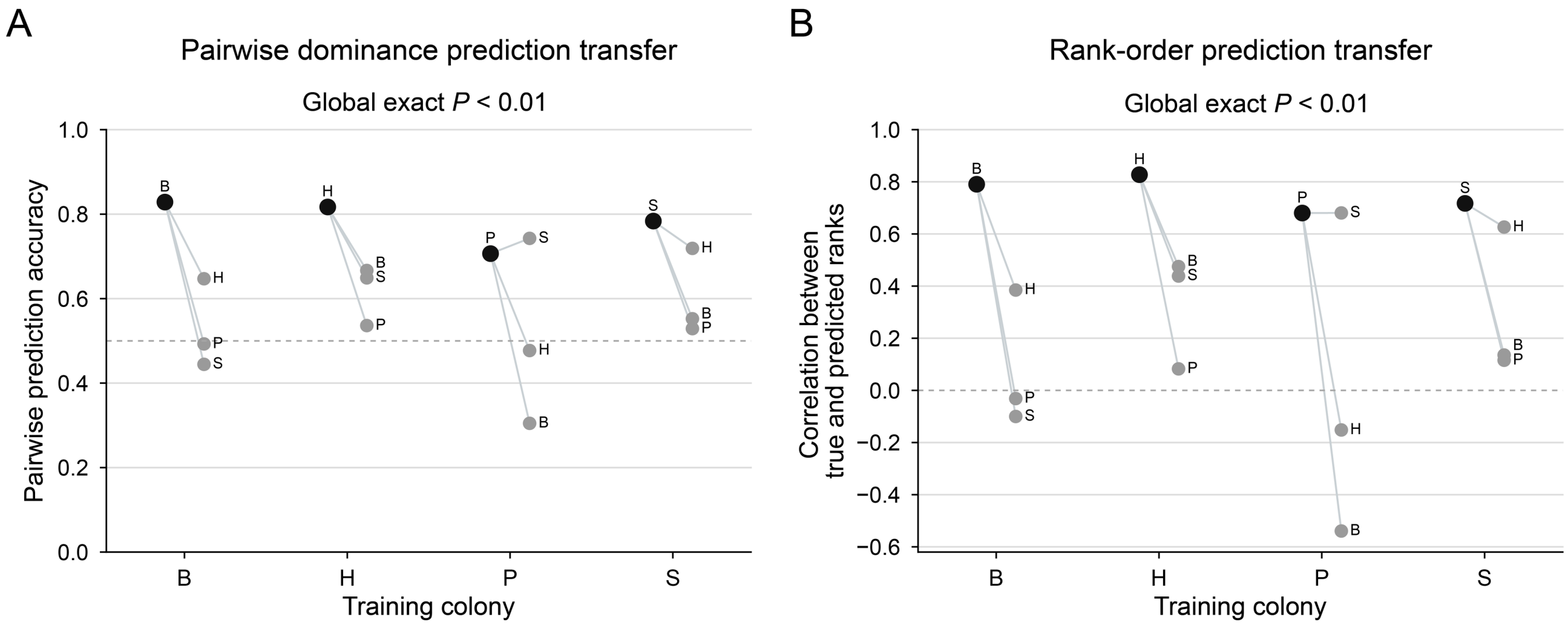

Supplementary Figure S7. Dominance and rank prediction models transfer poorly across colonies. (**A**) Pairwise dominance-prediction accuracy of PLS regression models fitted in each colony and evaluated within the same colony (black) or transferred to each of the other three colonies (gray). (**B**) Correlations between true and predicted rank orders. Letters identify the target colony, and lines connect predictions made by the same training-colony model. Same-colony performance exceeded cross-colony performance for both pairwise dominance prediction (mean accuracy, 0.78 versus 0.56; $P = 0.008$) and rank-order prediction (mean Spearman's *rho*, 0.75 versus 0.18; $P = 0.008$). P values were calculated using two-sided exact source-stratified permutation tests. Dashed lines indicate chance accuracy in A (50%) and zero correlation in B.
